# Manual control of catalytic reactions: Reactions by an apoenzyme gel and a cofactor gel

**DOI:** 10.1038/srep16254

**Published:** 2015-11-05

**Authors:** Yuichiro Kobayashi, Yoshinori Takashima, Akihito Hashidzume, Hiroyasu Yamaguchi, Akira Harada

**Affiliations:** 1 Department of Macromolecular Science, Graduate School of Science, Osaka University, Toyonaka, Osaka 560-0043, Japan; 2 Project Research Center for Fundamental Sciences, Graduate School of Science, Osaka University, Toyonaka, Osaka 560-0043, Japan

## Abstract

Enzymes play a vital role in catalysing almost all chemical reactions that occur in biological systems. Some enzymes must form complexes with non-protein molecules called cofactors to express catalytic activities. Although the control of catalytic reactions via apoenzyme–cofactor complexes has attracted significant attention, the reports have been limited to the microscale. Here, we report a system to express catalytic activity by adhesion of an apoenzyme gel and a cofactor gel. The apoenzyme and cofactor gels act as catalysts when they form a gel assembly, but they lose catalytic ability upon manual dissociation. We successfully construct a system with switchable catalytic activity via adhesion and separation of the apoenzyme gel with the cofactor gel. We expect that this methodology can be applied to regulate the functional activities of enzymes that bear cofactors in their active sites, such as the oxygen transport of haemoglobin or myoglobin and the electron transport of cytochromes.

Catalysts are one of the most important functional materials not only in organic chemistry but also in industry and in biological systems. The most common catalysts are enzymes. Many enzymes require an additional small molecule, known as a cofactor, to exhibit catalytic activities. For example, horseradish peroxidase (HRP) exhibits catalytic activity when L-histidine, at the active site of apohorseradish peroxidase (apoHRP), and iron porphyrin (FePor) form a complex via a metal–ligand interaction^1^. The catalytic activities cannot be expressed by apoHRP alone. One clear challenge in developing catalysts is the ability to control their activity on demand. Generally, catalytic activity can be controlled by pH^2^,^3^, temperature^4^,^5^, light^6^,^7^, electricity^8^, and ultrasound^9^,^10^. This requires specialized electrically powered devices that may be inconvenient for certain practical applications. In contrast, a controlled catalytic activity system, operated via mechanical forces, is versatile. However, there are few reports concerning catalytic activity via mechanical forces^11^.

Recently, several molecular-level phenomena have been expressed on a macroscopic level by using magnetic interactions^12^,^13^,^14^, electrostatic interactions^15^,^16^, hydrophile–lipophile balance^17^,^18^,^19^,^20^, and capillary effects^21^,^22^,^23^,^24^. Previously, we reported that the host-containing and guest-containing gels associate together to form the gel assembly^25^,^26^. Additionally, we have found that the gel that contained a FePor gel firmly adhere to the gel that contained L-histidine groups^27^. This result prompted us to investigate binding between the FePor and apoHRP gels. Moreover, if the FePor gel binds to the apoHRP gel, the gel assembly is expected to show catalytic behaviour for some substrates. In this particular case, the gel assembly shows the catalytic behaviour, and the gel assembly dissociation stops the catalytic reaction. Eventually, the catalytic reactions can be controlled, as the gel association and dissociation can be controlled using mechanical forces. Herein, we demonstrate that the apoHRP-containing gel adhere to the FePor-containing gel and resulted in an assembly. The gel assembly exhibit catalytic oxidation reactions for some substrates, such as peroxidase. Furthermore, when the gel assembly is separated by hand, individual gels did not exhibit catalytic behaviour (Fig. 1). This process was found to be repeatable.

## Results

### Preparation of the apoHRP and FePor gels

**Figure 1:**
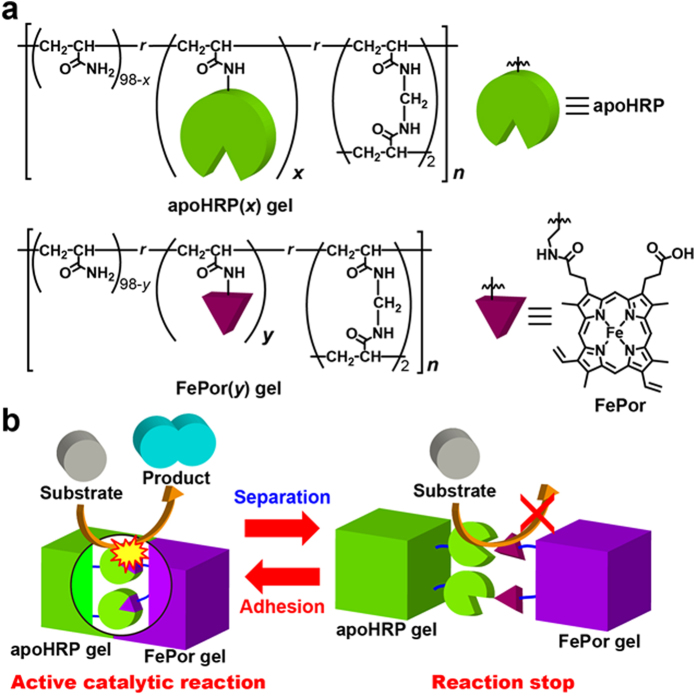
Chemical structures of the apoHRP(*x*) and FePor(*y*) gels. (**a**) ApoHRP and FePor moieties modified with the poly(acrylamide)-based gels. *x* and *y* indicate the mol% of the apoHRP and FePor moieties, respectively. (**b**) Switchable catalytic activity system via adhesion and separation of the apoHRP and FePor gels.

Polyacrylamide was selected as the main chain in this study because interactions of the -CONH groups with porphyrin or a protein are small when used in a gel electrophoresis. ApoHRP was prepared from HRP. HRP was dissolved in 100 mM sodium phosphate buffer (pH = 7.0). The pH of the solution was brought to pH = 1.9 by slowly adding 0.1 M HCl. The acidic HRP solution was extracted with 2-butanone to remove the heme, and it was then immediately neutralized with 1 M NaOH. The apoHRP in the neutral solution was obtained using dialysis with 50 mM sodium phosphate buffer (pH 7.0)^28^. FePor was prepared by mixing Por with FeCl_2_^27^. The apoHRP and FePor gels were prepared by modifying apoHRP and FePor to the acrylamide/acrylic acid/*N,N ′*-methylene bisacrylamide terpolymer gel (MBAAm, cross-linker; Scheme S1 and S2). The poly(acrylamide) gel (a blank gel) was prepared using radical copolymerization of acrylamide and MBAAm that was initiated by ammonium persulfate in aqueous solutions (Scheme S3). In all gels, the MBAAm unit was fixed to 2 mol%. UV-Vis spectroscopy characterized the functional monomeric unit content of the apoHRP and FePor gels. Figure 1 depicts the chemical structures of the apoHRP(*x*) and FePor(*y*) gels. Here, *x* and *y* represent the mol% of the apoHRP and FePor units, respectively. The *x* mol% content of apoHRP was determined to be 0.22, 0.80, or 1.0 mol% in the apoHRP(*x*) gels (Fig. S1). Similarly, the *y* mol% content of FePor was determined to be 0.47 or 1.0 mol% in the FePor(*y*) gels (Fig. S2).

### Interaction between the apoHRP and FePor units

**Figure 2:**
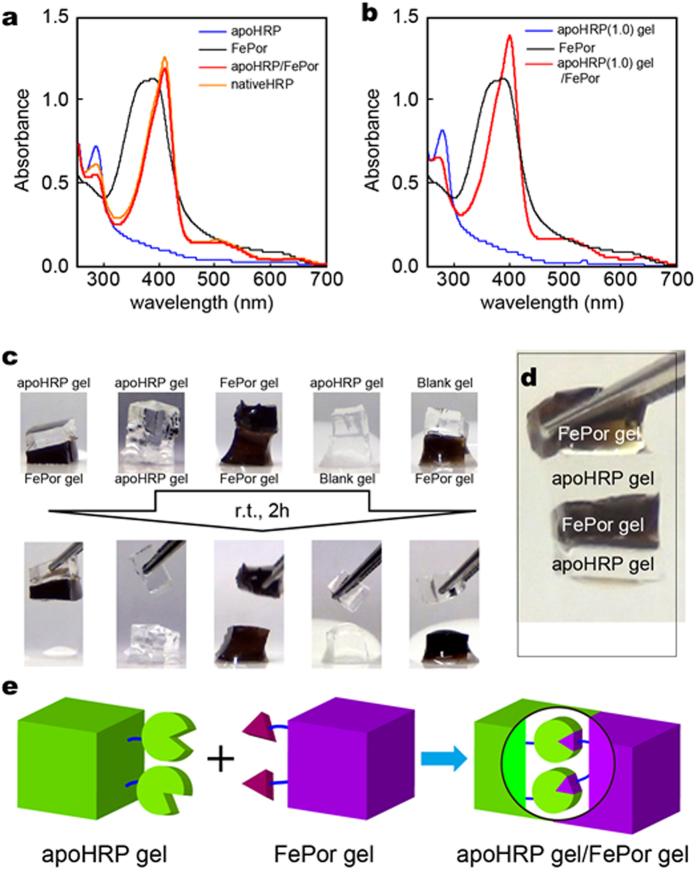
Complex formation of the apoHRP/FePor, apoHRP gel/FePor, and apoHRP gel/FePor gel. (**a**) UV-Vis spectra of apoHRP (blue, 35 μM), FePor (black, 20 μM), the apoHRP/FePor complex (red), and nativeHRP (orange, 38 μM) in 4% DMSO and 50 mM sodium phosphate buffer (pH 7.0). (**b**) UV-Vis spectra of the apoHRP(1.0) gel (blue), FePor (black, 20 μM), and the apoHRP(1.0) gel/FePor complex (red). (**c**) Adhesion experiments between functional hydrogels. The apoHRP(1.0) gel adheres to the FePor (1.0) gel, whereas the following combinations do not adhere to each other (the apoHRP(1.0) gel/apoHRP(1.0) gel, the FePor(1.0) gel/FePor(1.0) gel, the apoHRP(1.0) gel/blank gel, and the blank gel/FePor(1.0) gel). (**d**) Picture of the apoHRP (1.0) gel/FePor (1.0) gel assembly lifted via tweezers. (**e**) Schematic illustration of adhesion between the apoHRP and FePor gels via formation of the apoHRP/FePor complex.

Prior to the adhesion experiment between the apoHRP(*x*) and FePor(*y*) gels, the interaction between apoHRP and FePor was investigated in 4% DMSO and 50 mM sodium phosphate buffer (pH 7.0) (hereafter referred to as the buffer). Figure 2a shows the UV-Vis spectra of native HRP (orange), apoHRP (blue), FePor (black), and the apoHRP/FePor complex (red). Although the maximum absorption wavelength (λ_max_) of FePor showed λ_max_ = 398 nm, the λ_max_ of the apoHRP/FePor complex was similar to that of native HRP (λ_max_ = 410 nm). This red-shift is derived from complexation of L-histidine at active site of apoHRP and FePor by coordination bond^29^,^30^. The association constant (*K*_a_) of apoHRP with FePor was determined to be 3.8 × 10^6^ M^−1^ in buffer at 298 K (Fig. S3). These results indicate that FePor is properly accommodated in the heme pocket of apoHRP to form the apoHRP/FePor complex.

Based on the interaction between apoHRP and FePor, the complex formation of the apoHRP unit in an apoHRP(1.0) gel was investigated. The apoHRP(1.0) gel was immersed in a buffer with FePor (50 μM) (Scheme S5). The apoHRP(1.0) gel with FePor (apoHRP(1.0) gel/FePor complex) was washed with excess buffer. The maximum absorption band corresponding to FePor shifted from 394 nm to 400 nm in the UV-Vis spectra of the apoHRP(1.0) gel/FePor complex (Fig. 2b; Fig. S4, red). This result indicates that apoHRP in the apoHRP(1.0) gel forms a complex with FePor.

### Adhesion of the apoHRP gel to the FePor gel through molecular recognition

Next, we investigated the adhesion of the apoHRP gel(*x*) to the FePor gel(1.0). These gels were cut into *ca*. 5 mm × 5 mm × 2 mm cuboids using a razor. One of the gels was placed on top of the other, and the stack was left undisturbed for 2 h at r.t. under humid conditions. The apoHRP(1.0) gel adhered to the FePor(1.0) gel (Fig. 2c, Supplementary Movie 1). The apoHRP(1.0) gel/FePor(1.0) gel assembly was sufficiently strong that it could be lifted with tweezers (Fig. 2d, Supplementary Movie 2). We calculated the adhesion strength for the apoHRP(1.0) gel/FePor(1.0) gel assembly, using the adhesion area (25 mm^2^) and lifting of the gel weight (22 mg × 3), to be at least 28 Pa. In contrast, the same gel type (e.g., a combination of the apoHRP(1.0) gel/apoHRP(1.0) gel) and the combination of blank gel/apoHRP(1.0) gel and blank gel/FePor(1.0) gel did not adhere to each other. The apoHRP(1.0) gel adhered to the FePor(1.0) gel. However, the apoHRP (0.22 and 0.80) gel did not adhere to the FePor(1.0) gel (Fig. S5), and the apoHRP(1.0) gel did not adhere to the FePor(0.47) gel (Fig. S6). The adhesion strength of the assembled gels increased by increasing the molar content of apoHRP and FePor units in the gels, which is consistent with previous reports^25^,^26^,^27^. These results indicate that the adhesion between the apoHRP and FePor gels is due to complexation of apoHRP with the FePor unit on the gel surface (Fig. 2e).

Competitive experiments confirmed the formation of the gel assembly based on the complexation of apoHRP with FePor. The addition of apoHRP-containing buffer (350 μM) led to the dissociation of the apoHRP(1.0) gel/FePor(1.0) gel assembly (Fig. S7a, Supplementary Movie 3). This result is attributed to apoHRP in buffer competitively forming a complex with the FePor unit of the FePor(1.0) gel instead of the apoHRP unit in the apoHRP(1.0) gel because the concentration of apoHRP in buffer (1.4 μmol) is 1,500 times higher than in the apoHRP gel (0.95 pmol, Fig. S7b). These results indicate that the apoHRP/FePor complexation is responsible for the formation of the apoHRP(*x*) gel/FePor(*y*) gel assembly.

### Catalytic behaviour of the apoHRP/FePor complex

**Figure 3:**
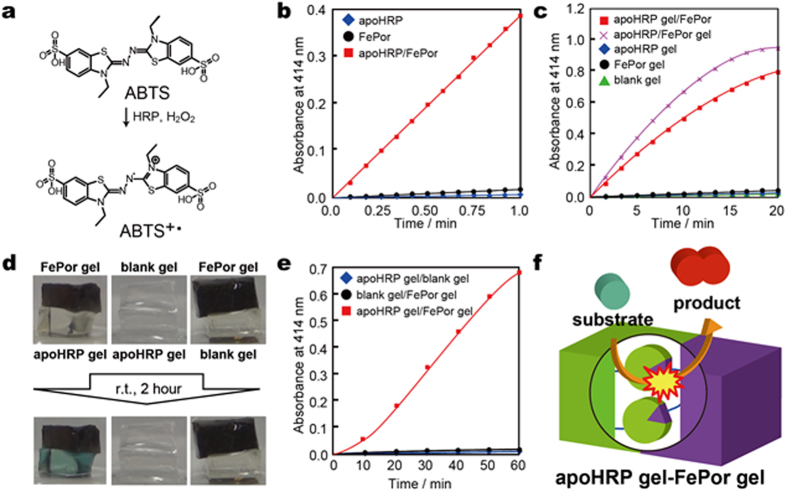
Expression of catalytic activity via adhesion of the apoHRP gel to the FePor gel. (**a**) Chemical structure of ABTS and its oxidation product (ABTS^+•^). (**b**) UV-Vis spectra of the oxidation of 0.5 mM ABTS in the presence of 1.0 μM apoHRP (blue diamonds), FePor (black dots), and the apoHRP/FePor complex (red squares) at 10 °C. (**c**) UV-Vis spectra of the oxidation of 0.5 mM ABTS in the presence of apoHRP (1.0) gel (blue diamonds), FePor (1.0) gel (black dots), blank gel (green triangles), the apoHRP (1.0) gel/FePor complex (red squares), and the apoHRP/FePor (1.0) gel complex (purple dots) at 10 °C. (**d**) Investigation of catalytic activity via adhesion of the apoHRP (1.0) gel/FePor (1.0) gel. (**e**) UV-Vis spectra of the oxidation of 0.5 mM ABTS in the presence of a combination of apoHRP (1.0) gel/blank gel (blue diamonds), blank gel/FePor (1.0) gel (black dots), and the apoHRP (1.0) gel/FePor (1.0) gel assembly (red squares). (**f**) Schematic illustration of the oxidation of ABTS via adhesion of the apoHRP gel to the FePor gel.

2,2′-Azino-bis(3-ethylbenzothiazoline-6-sulphonic acid) (ABTS) was used as a typical substrate oxidized by HRP (Fig. 3a). We investigated the oxidative catalytic activities of the apoHRP/FePor, apoHRP(*x*) gel/FePor, and apoHRP /FePor(*y*) gel complexes. The absorbance of the oxidized product of ABTS (ABTS^+•^) was measured in buffer using UV spectroscopy while stirring at 10 °C. Although ABTS was not oxidized by either apoHRP (Fig. 3b, blue diamonds) or FePor (Fig. 3b, black dots), the apoHRP/FePor complex (Fig. 3b, red squares) oxidized ABTS. The apoHRP/FePor complex oxidized other HRP substrates such as pyrogallol (Fig. S8a), 2-aminophenol (Fig. S8b), *o*-phenyldiamine (Fig. S8c), *o-*dianisidine (Fig. S8d), and hydroquinone (Fig. S8e). These results indicate that the apoHRP/FePor complex has the same catalytic system as native HRP. We investigated the catalytic activity of the apoHRP(1.0) gel/FePor complex and the apoHRP/FePor(1.0) gel complex for oxidizing ABTS. In addition, the oxidative reaction of ABTS in the presence of the apoHRP(1.0) gel/FePor complex and the apoHRP/FePor(1.0) gel complex was recorded using UV spectroscopy while stirring at 10 °C. The apoHRP(1.0) (Fig. 3c, blue diamonds), FePor(1.0) (Fig. 3c, black dots), and blank (Fig. 3c green triangles) gels did not cause oxidation of ABTS. However, the apoHRP(1.0) gel/FePor complex (Fig. 3c red squares) and the apoHRP/FePor(1.0) gel complex (Fig. 3c, purple crosses, Scheme S6) caused oxidation. The change in the UV-Vis spectra of ABTS in the presence of the apoHRP(1.0) gel/FePor complex was similar to that in the presence of the apoHRP/FePor complex (Fig. S9 and S10). These results indicate that the apoHRP and FePor units in the gels exhibited catalytic activity. An increase in the apoHRP unit molar content of the apoHRP(*x*) gel/FePor complex from 0.22 to 1.0 mol% increased the oxidation rate of ABTS (Fig. S11). This result indicates that the oxidation rate is based on the apoHRP unit molar content in the gel. Therefore, the oxidation rate can be regulated by changing the molar content of the apoHRP and FePor units in the gels.

### Catalytic behaviour due to adhesion between the apoHRP(*x*) and FePor(*y*) gels

As described above, catalytic activity exhibited upon the apoHRP and FePor complex formation. Based on these results, we investigated whether the apoHRP(1.0) gel and FePor(1.0) gel adhesion cause an oxidative catalytic reaction. The apoHRP(1.0) gel was soaked for 1 day in a buffer containing 0.5 mM ABTS and 5.0 mM H_2_O_2_. After absorbing ABTS and H_2_O_2_ into the apoHRP(1.0) gel, the FePor(1.0) gel was placed on top of the apoHRP(1.0) gel. Interestingly, as shown in Fig. 3d and in the Supplementary Movie 4, the blue oxidized product of ABTS appeared on the adhesive surface between the apoHRP(1.0) and FePor(1.0) gels. The combination of the blank gel/apoHRP(1.0) gel and the FePor(1.0) gel/blank gel did not show such colour change. We investigated the oxidative catalytic activity of the apoHRP(1.0)gel/FePor(1.0) gel assembly in a solution. The apoHRP(1.0) gel/FePor(1.0) gel was soaked in buffer containing 0.5 mM ABTS and 5.0 mM H_2_O_2_. The absorbance of the ABTS oxidized product in solution was measured at r.t. using UV-Vis spectroscopy. Fig. 3e shows the absorbance change in the oxidative reaction of ABTS in the presence of various gel combinations. The combination of apoHRP(1.0) gel/blank gel (Fig. 3e blue diamonds) and the blank gel/FePor(1.0) gel (Fig. 3e black dots) did not produce ABTS^+•^. However, the apoHRP(1.0) gel/FePor(1.0) gel assembly dramatically caused oxidation of ABTS (Fig. 3e red square). The same tendency was observed when pyrogallol was used as a substrate instead of ABTS (Fig. S12, S13; Supplementary Movie 5). The oxidation rate of ABTS in the presence of the apoHRP(1.0) gel/FePor(1.0) gel assembly depended on the substrate concentration; the oxidation rate in the presence of the apoHRP(1.0) gel/FePor(1.0) gel assembly increased when the ABTS concentration increased from 0.1 to 0.5 mM (Fig. S14). These results indicate that the apoHRP gel/FePor gel assembly has catalytic activity (Fig. 3f).

The oxidative rate was correlated with the adhesive surface area of the apoHRP(1.0) gel/FePor(1.0) gel assembly. The oxidative rate of ABTS in the presence of the apoHRP(1.0) gel/FePor(1.0) gel assembly increased when the adhesive area of the gel assembly increased from 6.2 to 25 mm^2^ (Fig. S15). This result indicated that the number of apoHRP with FePor complexes at the gel adhesive surface increased when the adhesive area of the apoHRP gel/FePor gel assembly increased.

Although it has been previously described, it is worth mentioning that the apoHRP(0.80) gel did not adhere to the FePor(1.0) gel and that the contact object of the apoHRP(0.80) gel/FePor(1.0) gel showed catalytic activity. A 5 g weight was placed on top of the contact object of the apoHRP gel/FePor gel to prevent dissociation of the contact object of the apoHRP gel/FePor gel in buffer (Fig. S16a). Fig. S18b shows the absorbance change in the oxidative reaction of ABTS in the presence of various gel concentrations. The contact object of the apoHRP(0.80) gel/FePor(1.0) gel oxidized ABTS in buffer, which suggests that the apoHRP and FePor units form complexes when gel surfaces contact without apoHRP gel/FePor gel adhesion.

The oxidative rate, in the presence of the contact object of the apoHRP(0.80) gel to the FePor(*y*) gel, increased when the molar content of the FePor unit increased from *y* = 0.47 to 1.0 mol% (Fig. S16b red squares and blue diamonds). However, the oxidative rate in the presence of contact object of the apoHRP(0.22) gel to the FePor(*y*) gel remained the same when the molar content of the FePor unit in the gel increased from *y* = 0.47 to 1.0 mol% (Fig. S16b black dots and purple crosses). This occurs because the apoHRP binding sites at the gel interface are fully occupied with FePor. These results indicate that by changing the gel size and molar content of the apoHRP and FePor units we can control the catalytic oxidation rate.

### Control of catalytic reactions

**Figure 4:**
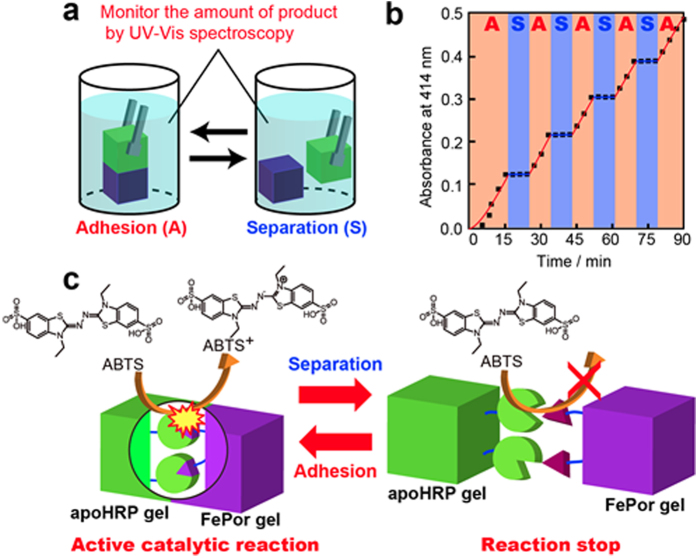
Switching the catalytic activity on and off via adhesion and separation of the apoHRP and FePor gels. (**a**) Component drawing of the experiment to switch the catalytic activity on and off via adhesion and separation of the apoHRP and FePor gels, respectively. (**b**) Oxidation of 0.5 mM ABTS in the presence of the apoHRP (0.80) gel/FePor (1.0) gel assembly (area A) and with separated gels (area S). (**c**) Schematic illustration of switching the catalytic activity on and off via adhesion of the apoHRP and FePor gels and their subsequent separation.

Additionally, we investigated the change in the production of ABTS oxidation product when the apoHRP(0.80) gel/FePor (1.0) gel was separated and manually re-adhered in buffer containing 0.5 mM ABTS and 5.0 mM H_2_O_2_ (Fig. 4a). Notably, separation of the apoHRP(0.80) gel/FePor(1.0) gel suspended ABTS oxidation (Fig. 4b blue area). However, upon readhesion of the apoHRP(0.80) and FePor(1.0) gels (Fig. 4b red area), the oxidation reaction accelerated. This process is repeatable. We obtained similar results using pyrogallol (Fig. S17). These results indicate that catalytic activity is switched on and off via adhesion and separation of the apoHRP and FePor gels, respectively (Fig. 4c).

## Discussion

We successfully constructed a system with controllable catalytic activity via the adhesion of gels at the macroscale. The oxidation of substrates for HRP is accelerated via adhesion of the polyacrylamide gel modified with apoHRP and FePor. The catalytic oxidation rate of these substrates is increased by increasing the adhesive area of the apoHRP gel/FePor gel assembly or the molar content of apoHRP and FePor in the gels. Thus, by changing the gel size or molar content of apoHRP or FePor in the gel, we can control the catalytic reaction. Furthermore, adhesion and separation of the apoHRP and FePor gels turns on and off the catalytic activity, respectively. We expect these findings to be applicable for various materials, such as drug carriers that release the drug upon gel adhesion.

## Methods

### General methods

The UV-vis absorption spectra were obtained using a JASCO V-650 spectrophotometer. Oxidation experiments with various substrates were performed in a solution containing 5.0 mM H_2_O_2_, 4% DMSO, and 50 mM sodium phosphate buffer (pH 7.0).

### Preparation of the apoHRP gel

Acrylamide (AAm), acrylic acid (AAc), and MBAAm were dissolved in 0.1 M NaOH. Upon the addition of ammonium persulfate and *N,N,N ′,N ′*-tetramethyl-1,2-ethanediamine to the monomer solution, the reaction mixture immediately became a gel. The gel was washed with water to remove unreacted monomers and initiators. Then, it was soaked in an excess of 0.1 M 2-(*N*-morpholino)ethanesulfonic acid (MES) buffer (pH 5.6). The carboxyl groups of the AAm-AAc-MBAAm gel were activated into sulfo-*N*-hydroxysulfosuccinimide-esters (sulfoNHS) in 10 mM 1-(3-dimethylaminopropyl)-3-ethylcarbodiimide hydrochloride and 20 mM *N*-hydroxysulfosuccinimide sodium salt for 10 h. Then, the sulfoNHS gel was washed with a 0.1 M MES buffer (pH 5.6) and was soaked for 14 h in a 0.1 M MES buffer containing apoHRP^28^. The resultant gel was thoroughly rinsed with a 0.1 M MES buffer (pH 5.6) and was soaked in excess buffer (Scheme S1). The mol% of apoHRP, *x*, was determined using the Lambert-Beer law from the absorbance of apoHRP in the gel with a thickness of 100 or 50 *μ*m (Fig. S1).

### Preparation of the FePor gel

AAm, MBAAm, and *N*-succinimidyl acrylate were dissolved in DMSO. After purging with dry argon for 3 h, 2-ketoglutaric acid was added to the monomer solution, which was subsequently irradiated using UV light with a wavelength of 365 nm for 8 h. The resultant gel was soaked in a DMSO solution containing FePor^26^. The FePor gel was washed with DMSO and was soaked in excess buffer (Scheme S2). The mol% of FePor, *y*, was determined using the Lambert-Beer law from the absorbance of FePor in the gel with a thickness of 10 *μ*m (Fig. S2). The UV-Vis spectra were obtained using a JASCO V-650 spectrophotometer.

### Switching of the catalytic activity

Substrate oxidation (2.0 mM pyrogallol or 0.5 mM ABTS) was examined in the presence of the apoHRP (0.80) gel–FePor (1.0) gel assembly and the separated apoHRP and FePor gels. The changes in absorbance of λ_max_ for the oxidation product of each substrate were monitored using a UV-Vis spectrometer.

## Additional Information

**How to cite this article**: Kobayashi, Y. *et al.* Manual control of catalytic reactions: Reactions by an apoenzyme gel and a cofactor gel. *Sci. Rep.* **5**, 16254; doi: 10.1038/srep16254 (2015).

## Supplementary Material

Supplementary Movie 1

Supplementary Movie 2

Supplementary Movie 3

Supplementary Movie 4

Supplementary Movie 5

Supplementary Information
