# Supplementary material for: Manual control of catalytic reactions: Reactions by an apoenzyme gel and a cofactor gel
Source: Sci Rep. 2015 Nov 5;5:16254. doi: 10.1038/srep16254 (PMC4633677; doi:10.1038/srep16254)
Supplement: Supplementary Information [file srep16254-s6.doc]

**Supplementary Information**

Manual control of catalytic reactions: Reactions by an apoenzyme gel and a cofactor gel

Yuichiro Kobayashi^1^, Yoshinori Takashima^1^, Akihito Hashidzume^1^, Hiroyasu Yamaguchi^1^, Akira Harada^1,2^*

^1^*Department of Macromolecular Science, Graduate School of Science, Osaka University, Toyonaka, Osaka 560-0043, Japan*

^2^*Project Research Center for Fundamental Sciences, Graduate School of Science, Osaka University, Toyonaka, Osaka 560-0043, (Japan)**To whom correspondence should be addressed. E-mail: harada@chem.sci.osaka-u.ac.jp (A.H.)

**Preparation of the apoHRP (*y*) gel**


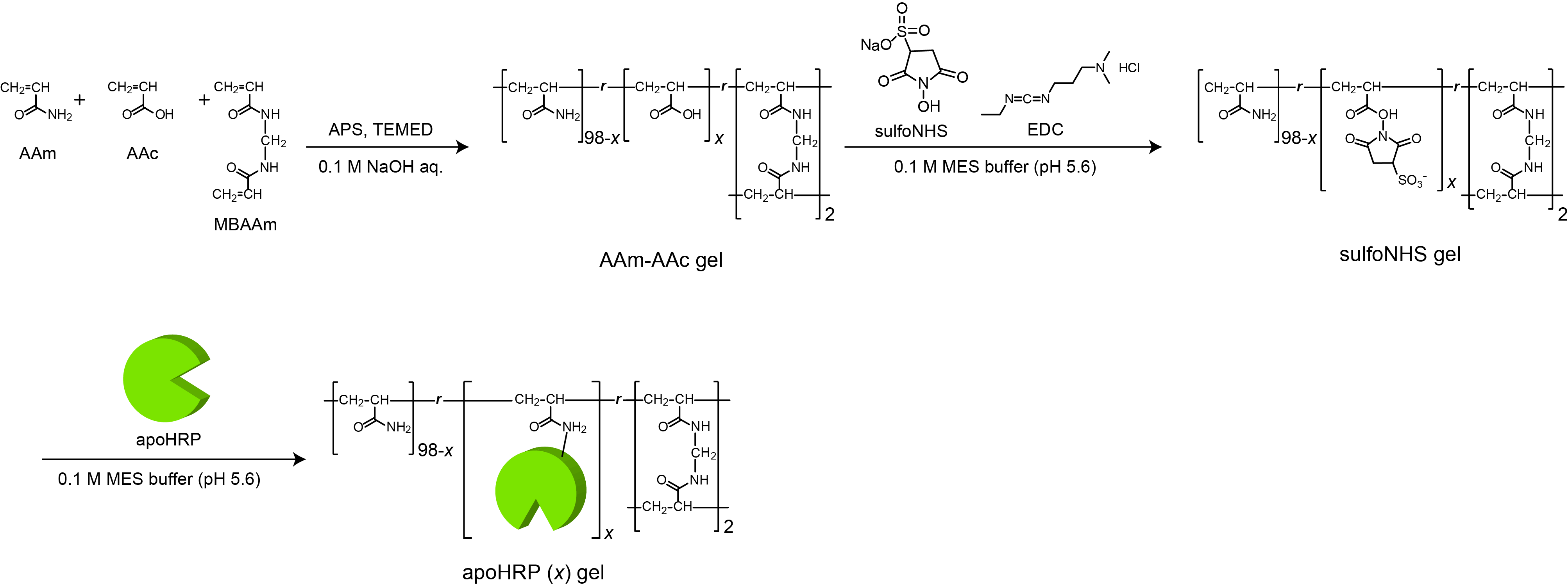
**Scheme S1.** Preparation of the apoHRP (*x*) gel.

**Preparation of the AAm–AAc gel**

Acrylamide (AAm) (0.29 g, 4.1 mmol), acrylic acid (AAc) (3.0 mg, 42 *μ*mol), and *N,N'*-methylenebis(acrylamide) (MBAAm) (13 mg, 83 *μ*mol) were dissolved in 0.1 M NaOH (2.0 mL). Upon the addition of ammonium persulfate (APS) (19 mg, 83 *μ*mol) and *N,N,N',N'*-tetramethyl-1,2-ethanediamine (TEMED) (12 *μ*L, 83 *μ*mol), the reaction mixture immediately became a gel. The gel was washed with water to remove the unreacted monomers and initiators and was subsequently soaked in an excess of 0.1 M 2-(*N*-morpholino)ethanesulfonic acid (MES) buffer (pH 5.6).

**Preparation of the sulfoNHS gel**

The reaction of 10 mM 1-(3-dimethylaminopropyl)-3-ethylcarbodiimide hydrochloride (EDC) and 20 mM *N*-hydroxysulfosuccinimide sodium salt (sulfoNHS) for 10 h converted the carboxyl group in the AAm–AAc gel into sulfo-NHS-ester. After the reaction, the gel was washed with 0.1 M MES buffer (pH 5.6).

**Preparation of the apoHRP (*x*) gel**

The reaction of sulfoNHS-esters in the sulfoNHS gel with the primary amine groups of apoHRP^1^ (35, 200, or 400 μM) for 14 h introduced apoHRP into the gel. The modified gel was thoroughly rinsed with a 0.1 M MES buffer (pH 5.6) and was subsequently soaked in an excess of solution containing 4% DMSO and 50 mM sodium phosphate buffer (pH 7.0). Additionally, apoHRP was prepared as previously described in the literature.

**Preparation of the FePor (*y*) gel**


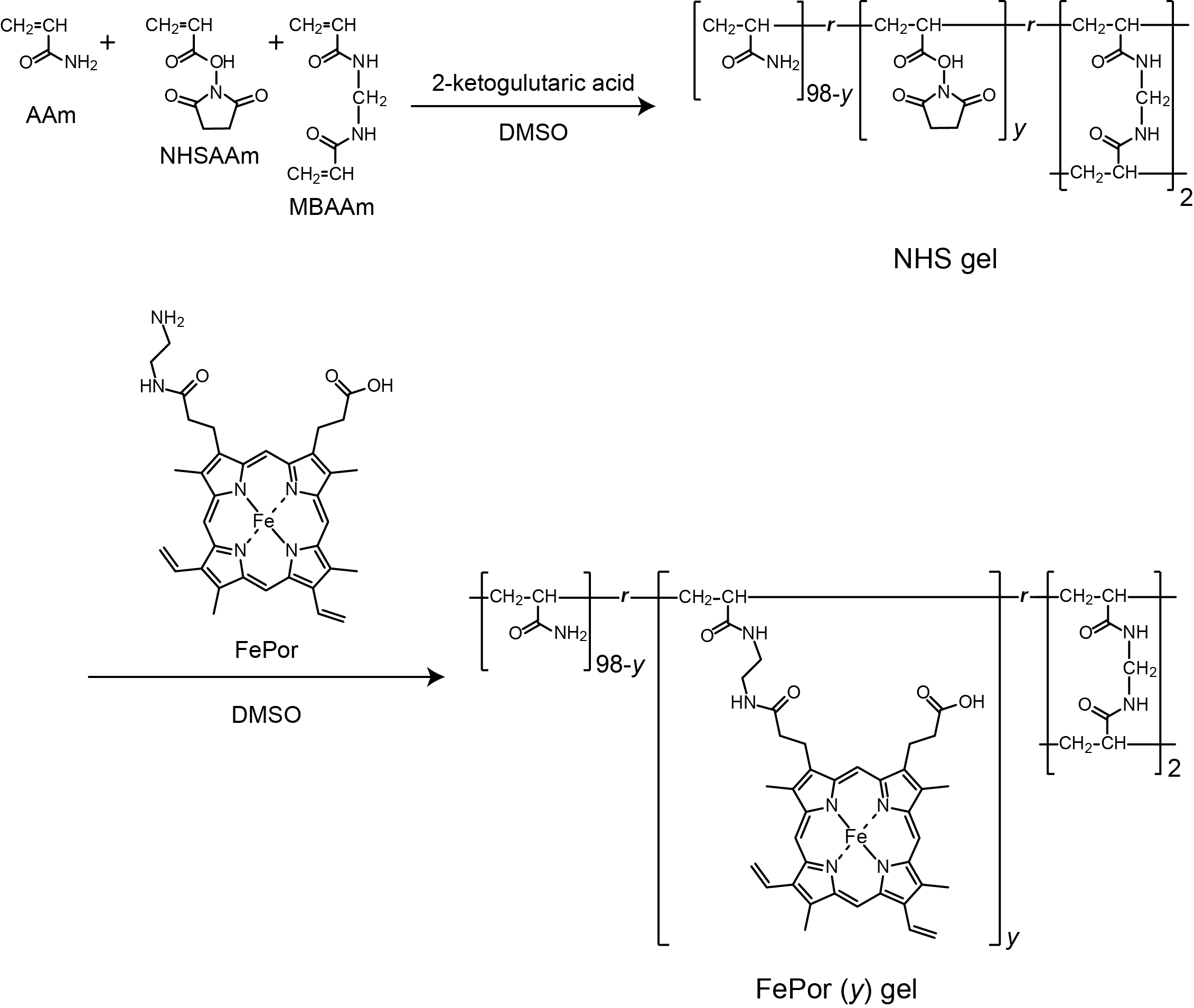
**Scheme S2.** Preparation of the FePor (*y*) gel.

AAm, MBAAm, and *N*-succinimidyl acrylate (NHSAAm) were dissolved in DMSO. After purging with dry argon for 3 h, 2-ketoglutaric acid was added to the monomer solution that was subsequently UV irradiated (365 nm) for 8 h. The gel was soaked in a DMSO solution (5 mL) of FePor (*3*). Then, the FePor gel was washed with DMSO and was subsequently soaked in an excess of the solution containing 4% DMSO and 50 mM sodium phosphate buffer (pH 7.0).

**Table S1.** Preparation of the FePor (*y*) gel


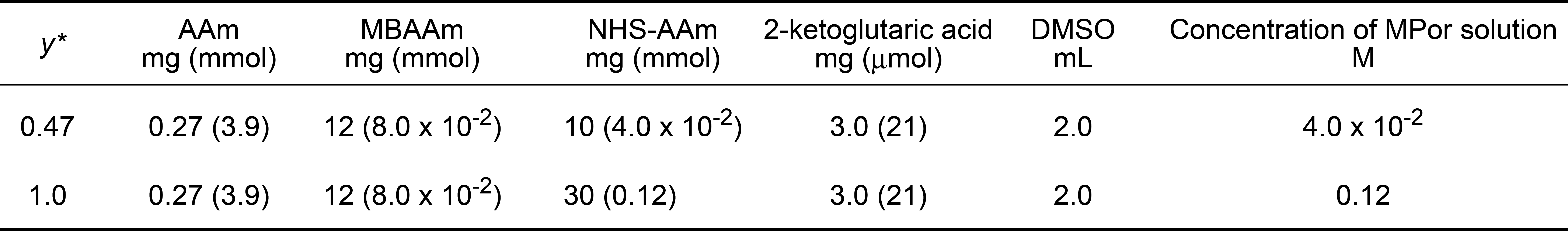
* *y* denotes the mol% of the FePor moiety

**Determination of the mol% of apoHRP in the gels**

The mol% of apoHRP in the gel was determined via λ_max_ at 278 nm (Fig. S1).

*A*_278 nm_: 0.78 (black), 1.4 (red), and 1.8 (blue)

*ε*_278 nm_: 2.0 × 10^4^ mol^-1^Lcm^-1^

l_apoHRP gel_ (thickness of the apoHRP gel): 100 and 50 *μ*m

The molar concentration of apoHRP in the gels: 4.0, 15, and 19 mM

The mol% of apoHRP gels: 0.22 (black), 0.80 (red), and 1.0 mol% (blue)


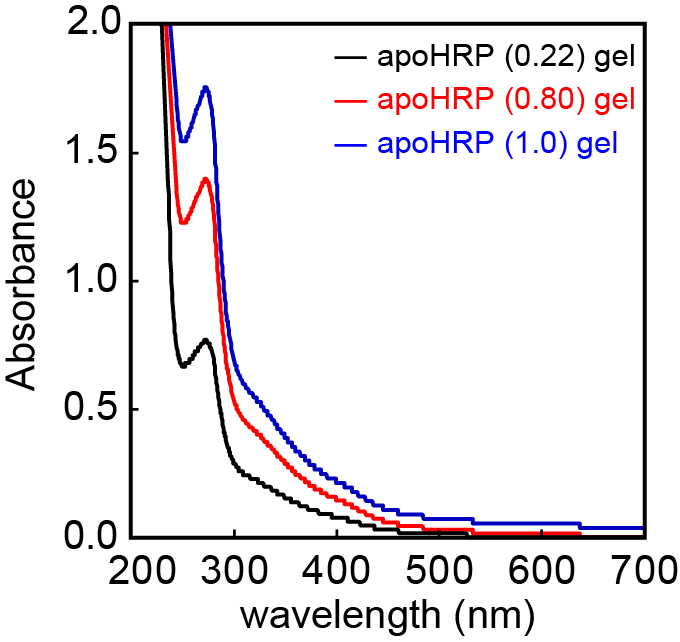


**Figure S1.** UV-Vis spectra of the apoHRP (*x*) gels, where *x* is 0.22 (black), 0.80 (red), and 1.0 (blue).

**Determination of FePor mol% in the gels**

The mol% of the FePor gel was determined using the same approach as for the apoHRP gel.

*A*_410 nm_: 0.059 (black) and 1.8 (red)

*ε*_410 nm_: 9.0 × 10^4^ mol^-1^Lcm^-1^

l_Fe-Por gel_: 10 *μ*m

The molar concentration of FePor in the gels: 9.4 and 20 mM

The mol% of FePor gels: 0.47 (black) and 1.0 mol% (red)


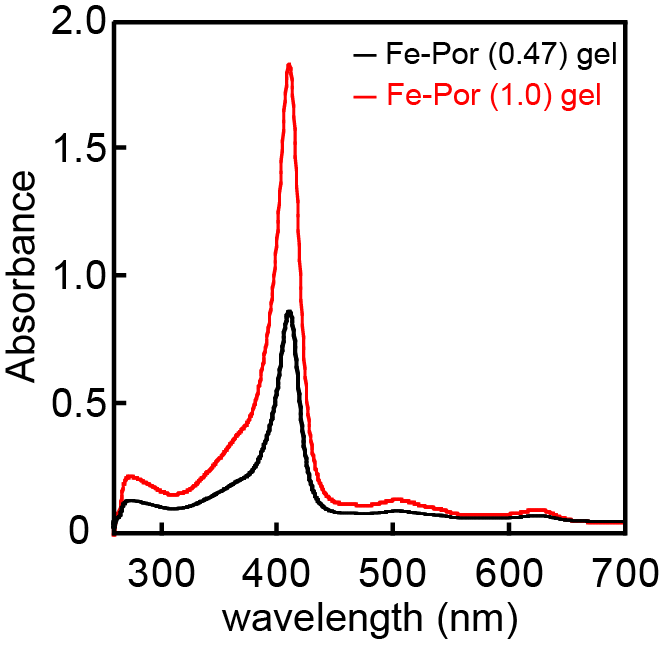


**Figure S2.** UV-Vis spectra of the FePor (*y*) gels before soaking in excess buffer, where *y* is 0.47 (black) and 1.0 (red).

**Preparation of the blank gel**


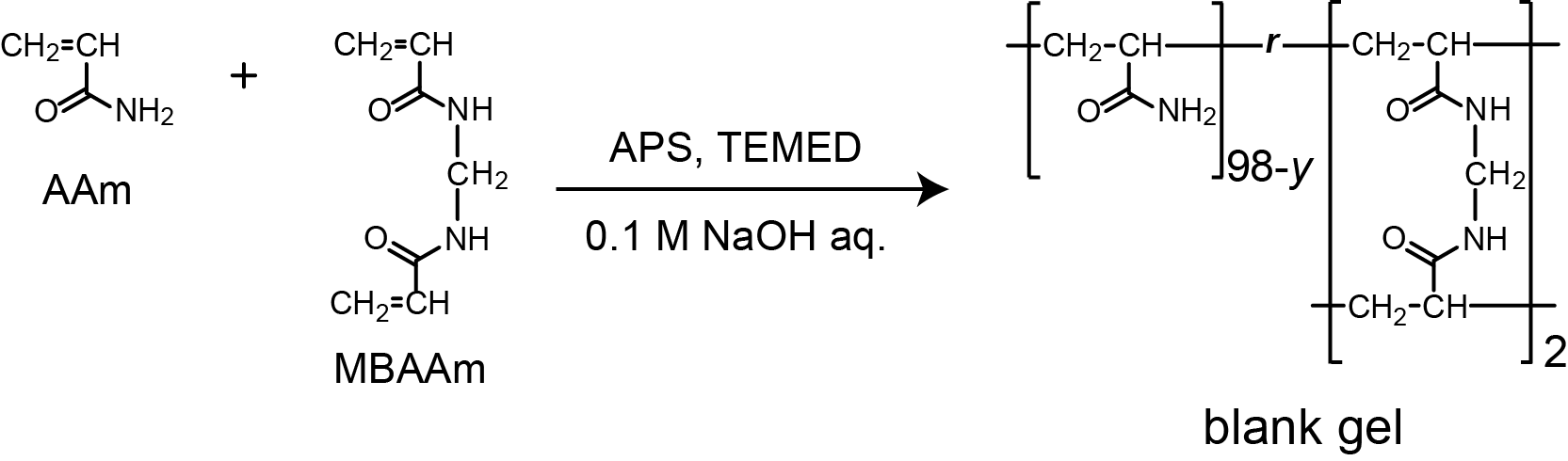
**Scheme S3.** Preparation of the blank gel.

AAm (0.29 g, 4.1 mmol) and MBAAm (13 mg, 83 *μ*mol) were dissolved in 0.1 M NaOH (2.0 mL). Upon the addition of APS (19 mg, 83 *μ*mol) and TEMED (12 *μ*L, 83 *μ*mol), the reaction mixture immediately became a gel. The gel was washed with water to remove the unreacted monomers and initiators and was subsequently soaked in excess solution containing 4% DMSO and a 50 mM sodium phosphate buffer (pH 7.0).

**Preparation of the apoHRP/FePor complex**


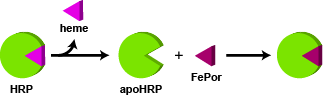


**Scheme S4.** Complex formation between apoHRP and FePor.

ApoHRP was prepared according to the procedure used by Hayashi et al^2^. The following method was used to prepare apoHRP/FePor. The FePor solution was added dropwise to the apoHRP solution while gently shaking the solution in an ice bath. After slowly shaking the solution for 30 min at 4 °C, the mixture was immediately concentrated using ultrafiltration (MWCO 10,000) to remove the low molecular weight materials. Complexation between apoHRP and FePor was monitored using UV-Vis spectroscopy.

**Determination of the association constant between apoHRP and FePor**

Figure S3 shows the UV-Vis spectral changes observed upon the addition of apoHRP at r.t. The Soret band of FePor shifted from 398 nm to 410 nm as the absorbance increased (Fig. S3a). Figure 3b plots the absorbance difference (Δ*A*_410nm_) against *C*_apoHRP_, which is fitted using eq. S1. This result indicates that the interaction between apoHRP and FePor can be analysed based on the formation of a one-to-one complex. The association constant (*K*) was determined to be 3.8 × 10^6^ M^-1^.

l = optical path length

Δ*ε* = *ε*_apoHRP-FePor_ *ε*_FePor_


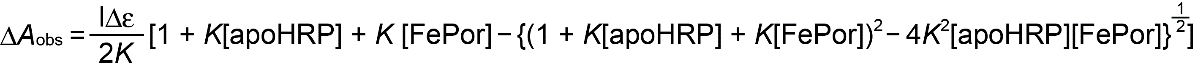
 (S1)


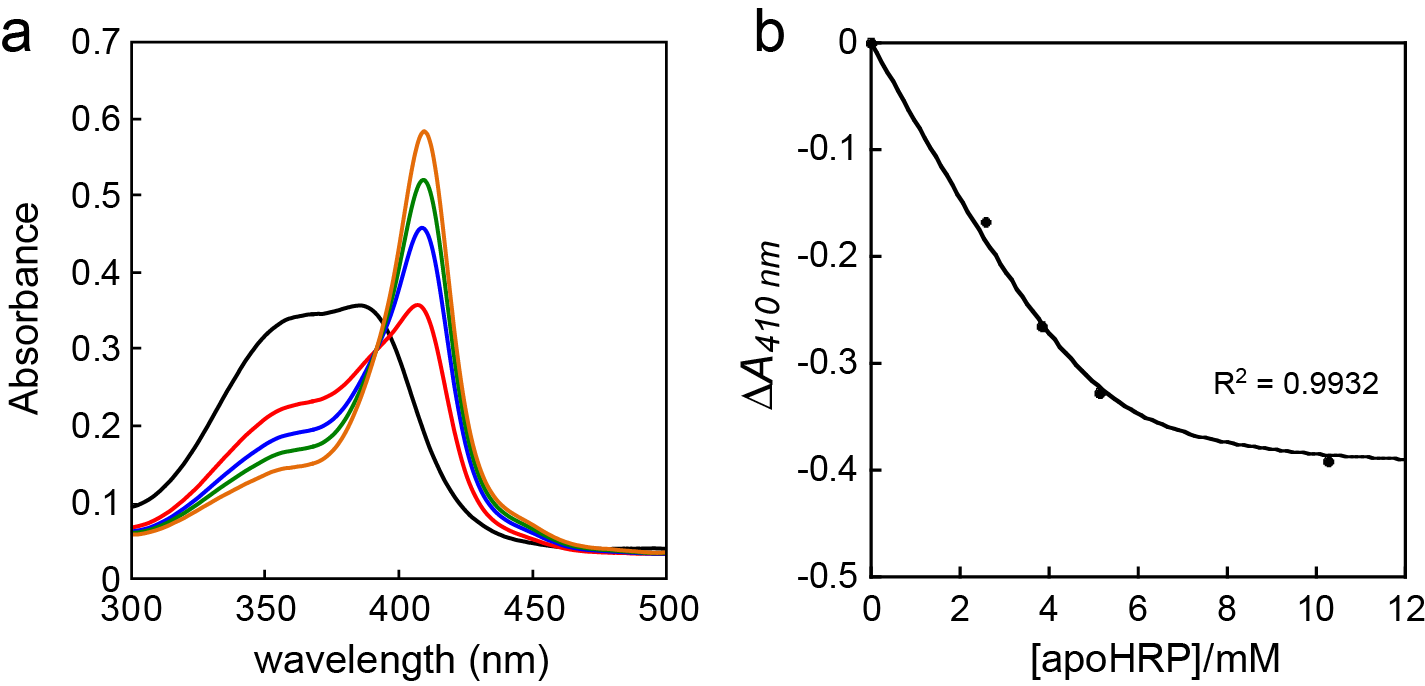
**Figure S3.** **a** UV-Vis spectral changes of FePor (5.13 μM black) upon addition of apoHRP [2.56 (red), 3.84 (blue), 5.13 (green), and 10.3 μM (orange)] to the solution at r.t. **b** Intensity difference at 410 nm of FePor (Δ*A*_410 nm_) as a function of *C*_apoHRP_. Additionally, the best-fitted curve using eq. S1 is demonstrated.

**Preparation of the apoHRP(*x*) gel/FePor complex**


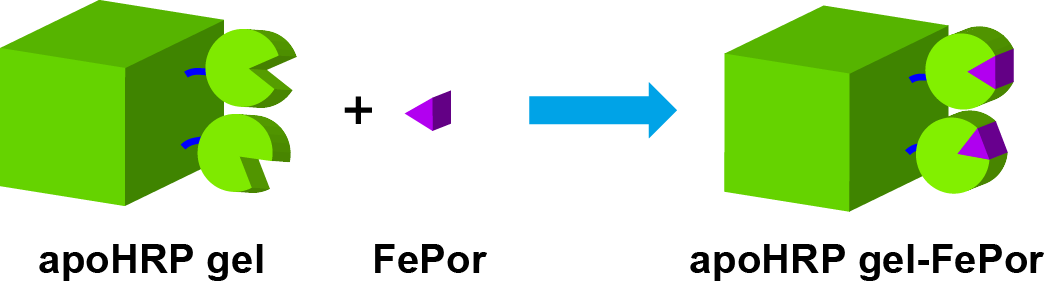


**Scheme S5.** Preparation of the apoHRP(*x*) gel/FePor complex

The apoHRP(1.0) gel /FePor complex was prepared using the following method.

The apoHRP(1.0, 0.80, or 0.22) gel (5 × 5 × 2 mm) was soaked in a FePor solution (50, 40, or 10 μM) containing 4% DMSO and 50 mM sodium phosphate buffer (pH 7.0). Then, the gel was rinsed with a solution containing 4% DMSO and 50 mM sodium phosphate buffer (pH 7.0) to yield the apoHRP gel/FePor complex. These apoHRP gel/FePor complexes were cut and placed between the quartz boards (Fig. S4) and were measured using UV-Vis spectroscopy.


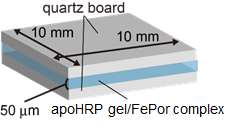


**Figure S4.** Illustration of sample used for UV-Vis spectroscopy.

**Adhesion test of the apoHRP(*x*) and FePor(1.0) gels.**

The apoHRP(*x*) and FePor(1.0) gels were cut into *ca*. 5 mm × 5 mm × 2 mm cuboids using a razor. The apoHRP(*x*) gel was placed on top of the FePor(1.0) gel, and the stack was left undisturbed for 2 h at r.t. under humid conditions. The apoHRP(1.0) gel adhered to the FePor(1.0) gel (Fig. S5c). However, the apoHRP(0.22 and 0.80) gel did not adhere to the FePor(1.0) gel (Fig. S5a, b).


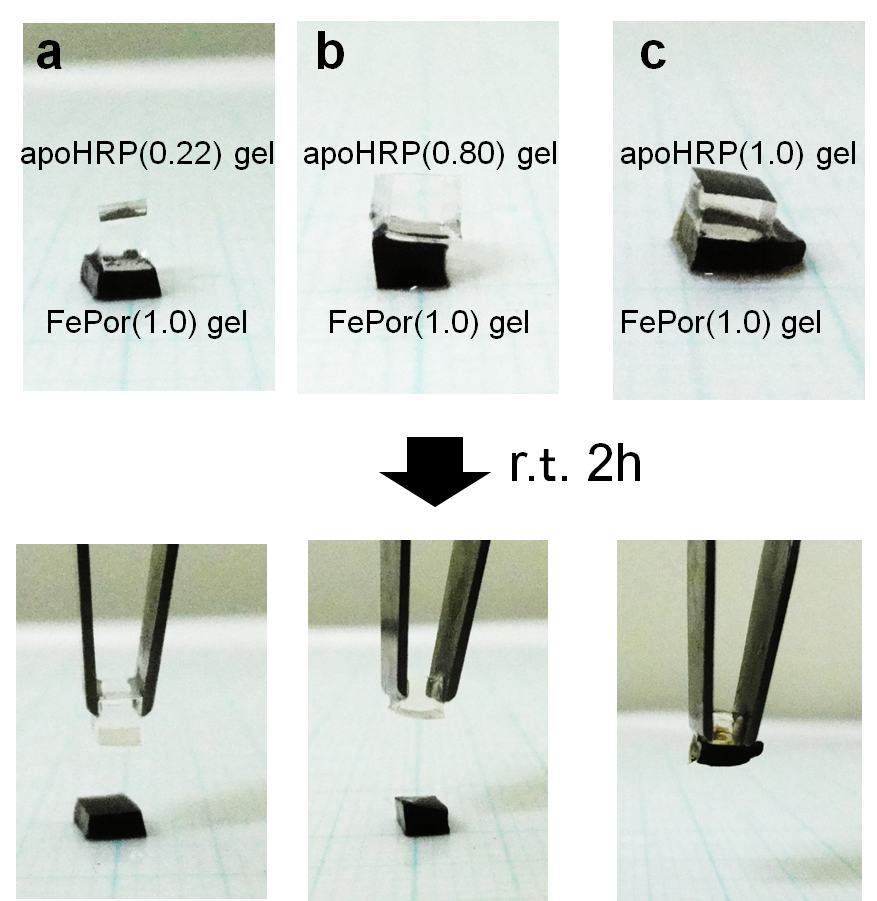


**Figure S5.** Differences in adhesion behaviour due to differences in the mole content of apoHRP in the apoHRP gel. a; the apoHRP(0.22) and FePor (1.0) gels, b; the apoHRP(0.80) and FePor (1.0) gels c; the apoHRP(1.0) and FePor (1.0) gels.

**Adhesion test of the apoHRP(1.0) and FePor(*y*) gels.**

The apoHRP(1.0) and FePor(*y*) gels were cut into *ca*. 5 mm × 5 mm × 2 mm cuboids using a razor. The apoHRP(1.0) gel was placed on top of the FePor(*y*) gel, and the stack was left undisturbed for 2 h at r.t. under humid conditions. The apoHRP(1.0) gel adhered to the FePor(1.0) gel (Fig. S6b). However, the FePor(0.47) gel did not adhere to the apoHRP(1.0) gel (Fig. S6a).


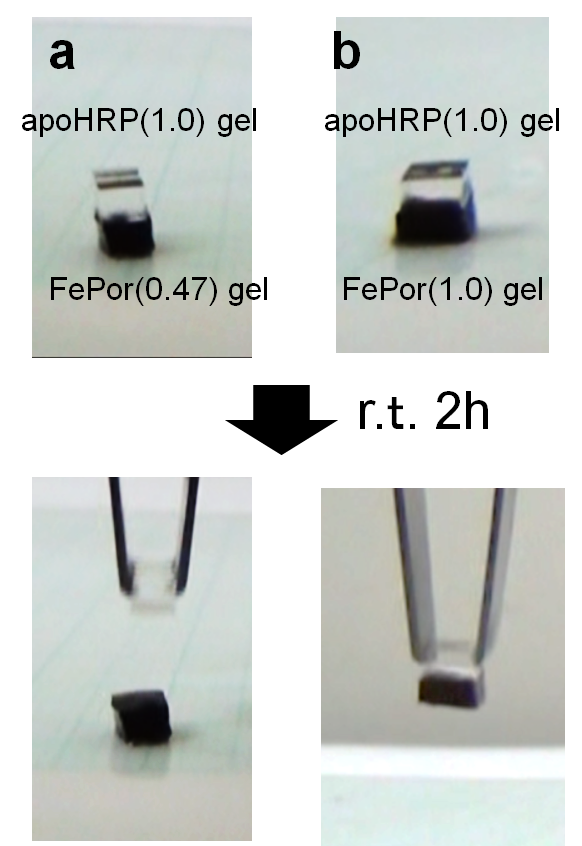


**Figure S6.** Differences in adhesion behaviour due to differences in the mole content of FePor in the FePor gel. **a**; the apoHRP(1.0) and FePor (0.47) gels, **b**; the apoHRP(1.0) and FePor (1.0) gels.

**Competitive experiments of the apoHRP gel/FePor gel assembly**

The apoHRP(1.0) gel/FePor(1.0) gel assembly was agitated in 25 mL of solution containing 4% DMSO, 50 mM sodium phosphate buffer (pH 7.0), and a solution containing 350 μM apoHRP, 4% DMSO, and 50 mM sodium phosphate buffer (pH 7.0) at 1000 rpm using EYELA CUTE MIXER CM-1000 at r.t. Agitation of the gels in a 350 μM apoHRP-containing solution caused the apoHRP(1.0) gel/FePor(1.0) gel assembly to dissociate (Figs. S8a, b; Supplementary Movie 3).


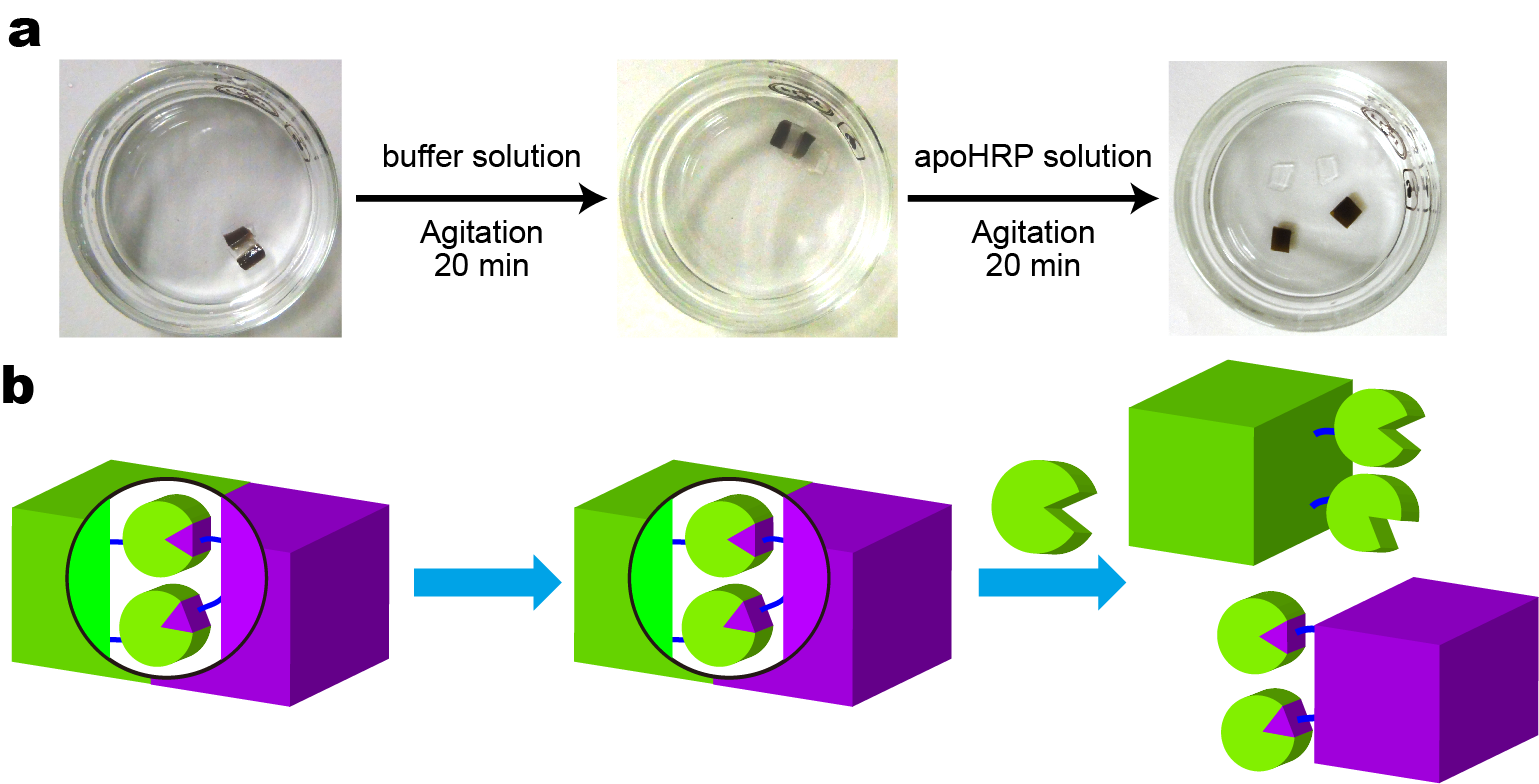


**Figure S7.** **a** The apoHRP (1.0) gel/FePor (1.0) gel assembly did not dissociate in a solution containing 4% DMSO and 50 mM sodium phosphate buffer (pH 7.0). However, replacing the solution with a solution containing 350 μM apoHRP, 4% DMSO, and 50 mM sodium phosphate buffer (pH 7.0) led to the dissociation of the apoHRP (1.0) gel/FePor (1.0) gel assembly. **b** Schematic illustration of the dissociation of the apoHRP (1.0) gel/FePor (1.0) gel assembly in the presence of apoHRP.

**Oxidation of various substrates in the presence of the apoHRP/FePor complex**

We investigated the oxidation of substrates in the presence of apoHRP, FePor, and the apoHRP/FePor complex. The oxidized product was recorded using UV spectroscopy in 0.5 min intervals (Fig. S10a). Purpurogallin, which is produced via oxidation of pyrogallol, was recorded at λ_max_ = 420 nm. Similarly, oxidation for other substrates was examined under the same oxidizing condition of ABTS and pyrogallol (Fig. S10b–e).


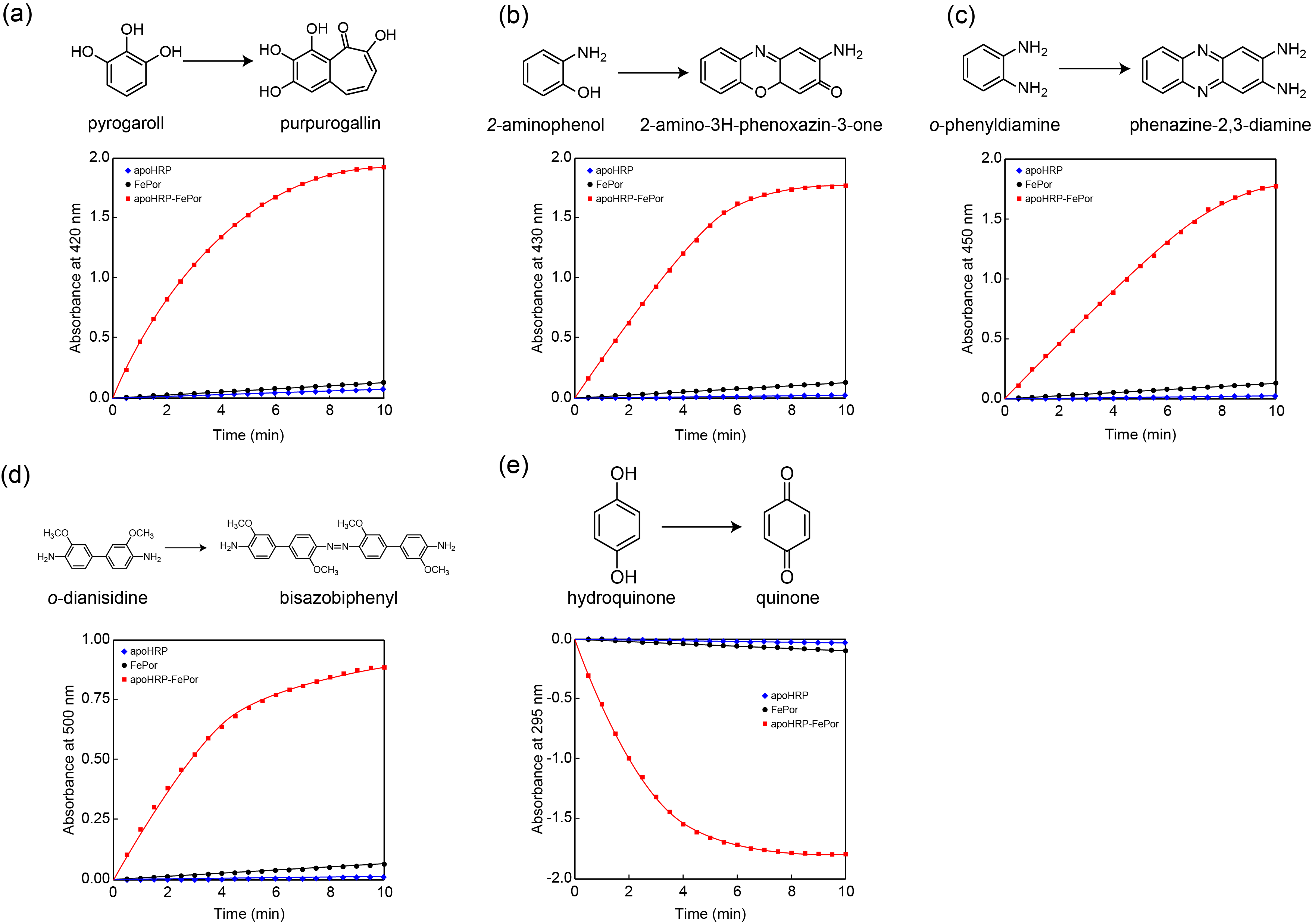
**Figure S8**. Oxidation of **a** 2.0 mM pyrogallol, **b** 3.0 mM 2-aminophenol, **c** 3.0 mM *o*-phenyldiamine, **d** 0.41 mM *o*-dianisidine, and **e** 1.2 mM hydroquinone in the presence of 1.0 μM apoHRP (blue diamonds), FePor (black circles), and 1.0 μM apoHRP-FePor (red squares). These experiments were performed in a solution containing 4% DMSO and 50 mM sodium phosphate buffer (pH 7.0) at 10 ºC.

**Preparation of the apoHRP/FePor(1.0) gel complex**


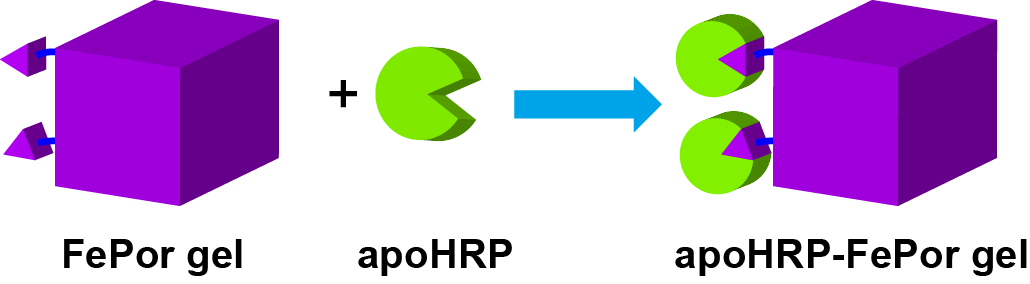
**Scheme S6.** Preparation of the apoHRP-FePor gel

The apoHRP/FePor(1.0) gel complex was prepared using the following method.

The FePor (1.0) gel (5 × 5 × 2 mm^3^) was soaked in the apoHRP solution (40 μM) for 12 h. After the prescribed time, the crude apoHRP/FePor(1.0) gel complex was rinsed with an excess of a solution containing 4% DMSO and 50 mM sodium phosphate buffer (pH 7.0) several times to give the apoHRP/FePor(1.0) gel complex.

**Comparison of apoHRP–FePor with apoHRP gel–FePor for oxidizing various substrates**

The oxidation products for each substrate in the presence of apoHRP–FePor (Fig. S9 black) or apoHRP gel–FePor (Fig. S9 blue) in a solution containing 5.0 mM H_2_O_2_, 4% DMSO, and 50 mM sodium phosphate buffer (pH 7.0) were monitored using UV-Vis spectroscopy to confirm that the reaction mechanism of apoHRP-FePor and apoHRP gel-FePor is same. The reaction temperature was set at 10 °C.


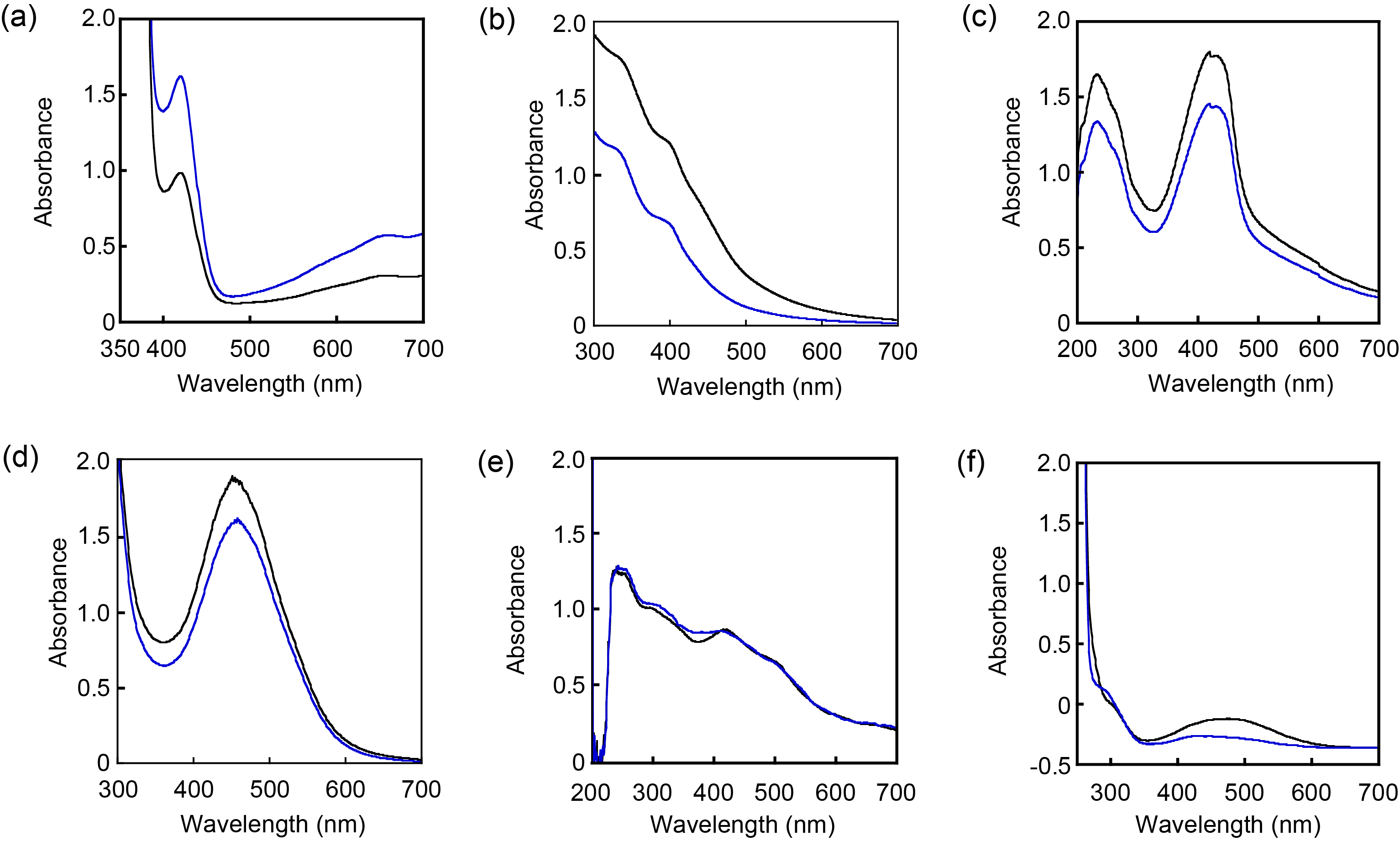


**Figure S9.** Oxidation of **a** ABTS, **b** pyrogallol, **c** 2-aminophenol, **d** *o*-phenyldiamine, **e** *o*-dianisidine, and **f** hydroquinone in the presence of apoHRP–FePor (black) or apoHRP gel–FePor (blue). These experiments were performed in a solution containing 4% DMSO and 50 mM sodium phosphate buffer (pH 7.0) at 10 ºC.

**Oxidation of various substrates in the presence of apoHRP gel–FePor**

Similar to the measurements used for apoHRP–FePor, oxidation of the substrates for HRP was examined in the presence of the apoHRP gel, blank gel, and apoHRP gel–FePor (Fig. S10).


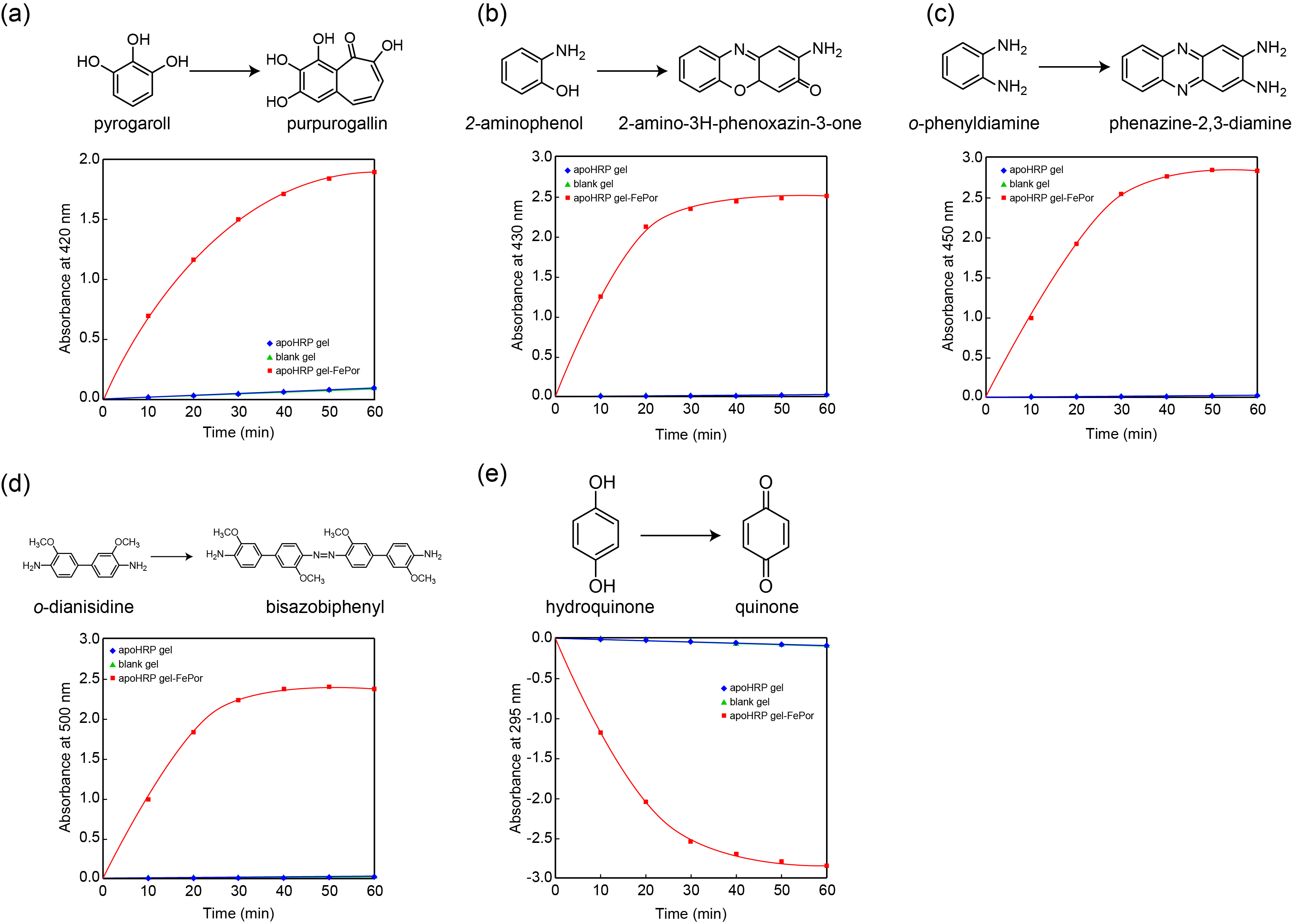


**Figure S10**. Oxidation of **a** 2.0 mM pyrogallol, **b** 3.0 mM 2-aminophenol, **c** 3.0 mM *o*-phenyldiamine, **d** 0.41 mM *o*-dianisidine, and **e** 1.2 mM hydroquinone in the presence of the apoHRP gel (blue diamonds), blank gel (green triangles), and apoHRP(1.0) gel–FePor (red squares). These experiments were performed in a solution containing 4% DMSO and 50 mM sodium phosphate buffer (pH 7.0) at 10 ºC.

**Oxidation of ABTS in the presence of apoHRP gel–FePor with different mol% of apoHRP in the gel**

Similar to apoHRP–FePor, the oxidation of ABTS with the apoHRP(0.22, 0.80, or 1.0) gel/FePor complex was examined.


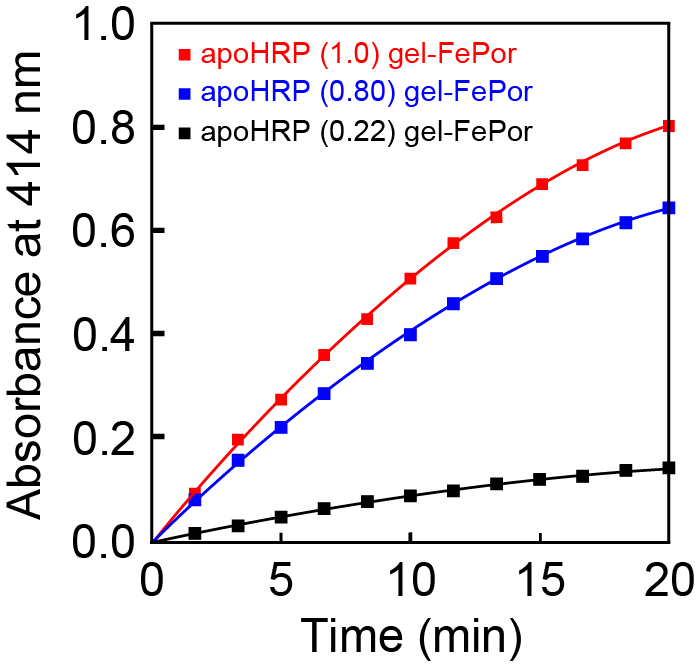


**Figure S11**. Oxidation of 0.5 mM ABTS in the presence of apoHRP(1.0) gel/FePor (red), apoHRP(0.80) gel/FePor (blue), and apoHRP(0.22) gel/FePor (black). These experiments were performed in a solution containing 4% DMSO and 50 mM sodium phosphate buffer (pH 7.0) at 10 ºC.

**Investigation of the catalytic activity via adhesion of the apoHRP and FePor gels**

The ApoHRP(1.0) gel was soaked in 50 mM sodium phosphate buffer (pH 7.0) containing 2.0 mM pyrogallol and 5.0 mM H_2_O_2_ for 2 h. When the FePor(1.0) gel was placed on top of the apoHRP(1.0) gel, a brown product ascribed to oxidation of pyrogallol appeared on the adhesive surface of the apoHRP(1.0) and FePor(1.0) gels. Although the combination of blank gel/apoHRP(1.0) gel and FePor(1.0) gel/blank gel gave the colour change to light brown due to self-oxidation of pyrogallol, the concentration of the reaction product was very low. On the other hand, the apoHRP(1.0) gel/FePor(1.0) gel assembly was found to accelerate the oxidation of pyrogallol to give the high concentration of products. These results indicated that the apoHRP(1.0) gel/FePor(1.0) gel assembly accelerated oxidation of pyrogallol.


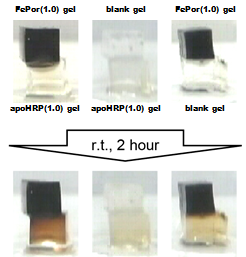


**Figure S12**. Investigation of catalytic activity via adhesion of the apoHRP(1.0) gel/FePor(1.0) gel.

**Oxidation of pyrogallol via the apoHRP gel/FePor gel assembly**

The oxidation of pyrogallol via the apoHRP(1.0) gel/FePor(1.0) gel assembly was examined in the same manner as ABTS. The combination of apoHRP(1.0) gel/blank gel (Fig. S13 blue diamond) and FePor(1.0) gel/blank gel (Fig. S13 black circle) gave a small amount of the self-oxidation product, however, the apoHRP(1.0) gel/FePor(1.0) gel assembly (Fig. S13 red square) dramatically accelerated oxidation of pyrogallol.


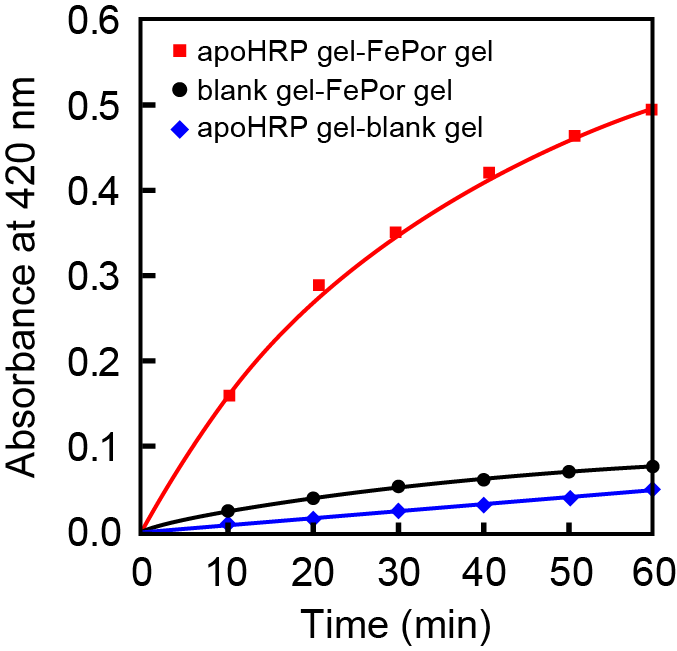


**Figure S13**. Oxidation of 2.0 mM pyrogallol in the presence of the following combinations: apoHRP(1.0) gel/blank gel (blue diamonds), blank gel–FePor(1.0) gel (black circles), and the apoHRP(1.0) gel/FePor(1.0) gel assembly (red squares).

**Dependence of catalytic activity on the substrate concentration in the apoHRP gel/FePor gel assembly**

The apoHRP(1.0) gel/FePor(1.0) gel assembly was soaked in 50 mM sodium phosphate buffer (pH 7.0) containing 5.0 mM H_2_O_2_ and ABTS (0.1, 0.25, and 0.5 mM). ABTS oxidation was observed using UV-Vis spectroscopy. The oxidation rate increased when the ABTS concentration increased from 0.1 to 0.5 mM (Fig. S14 blue diamonds and red squares, respectively).


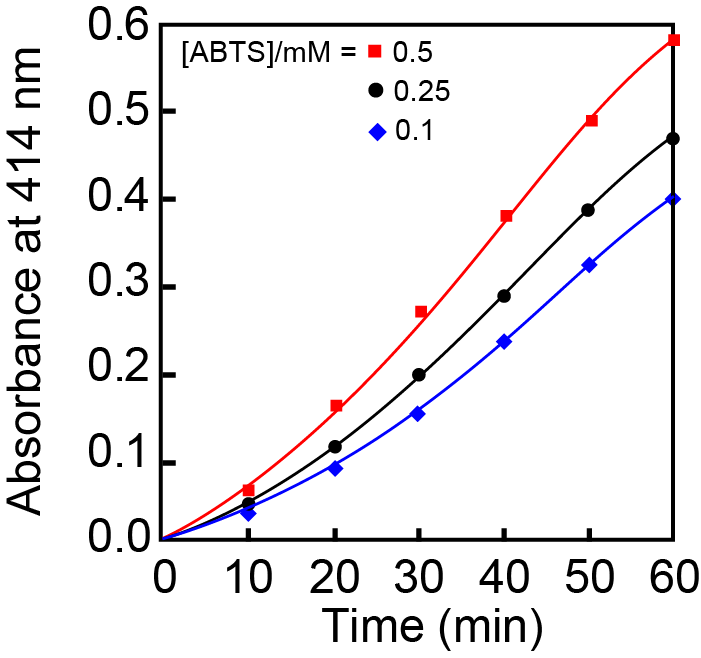


**Figure S14**. Oxidation of 0.1 (blue diamonds), 0.25 (black circles), and 0.5 (red squares) mM ABTS in the presence of the apoHRP(1.0) gel/FePor(1.0) gel.

**Oxidation of ABTS in the presence of apoHRP(1.0) gel/FePor(1.0) gel assemblies with different adhesive areas.**

Various sizes of apoHRP(1.0) gels (2.5 mm × 2.5 mm × 2 mm, 5 mm × 2.5 mm × 2 mm, and 5 mm × 5 mm × 2 mm) were stacked on top of the FePor(1.0) gel (5 mm × 5 mm × 2 mm). The ABTS oxidation rate increased as the adhesive area of the assembly increased from 6.2 to 25 mm^2^.


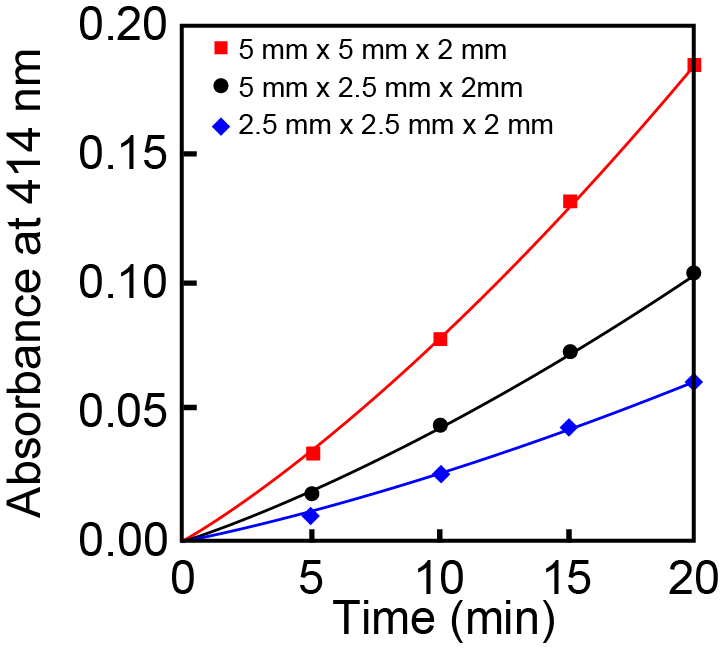


**Figure S15**. Oxidation of 0.5 mM ABTS in the presence of apoHRP(1.0) gel**/**FePor(1.0) gel assemblies with various sizes: 5 mm × 5 mm × 2 mm (red squares), 5 mm × 2.5 mm × 2 mm (black circles), and 2.5 mm × 2.5 mm × 2 mm (blue diamonds).

**Oxidation of ABTS in the presence of apoHRP gel/FePor gel with different mol% of apoHRP or FePor in the gel.**

ApoHRP gels with various mol% (0.22 and 0.80) and FePor gels (0.47 and 1.0) were allowed to come into contact with each other. The combination of apoHRP(*x*) gel/FePor(*y*) gel exhibited catalytic activity when a 5-g weight was placed on top of the combination of apoHRP (*x*) gel/FePor (*y*) gel (Fig. 16a). The ABTS oxidation rate increased as the concentration of FePor increased from 0.47 to 1.0 mol% in the apoHRP(0.80) gel/FePor(*y*) gel combination (Fig. S16b red squares and blue diamonds). However, the ABTS oxidation rate did not change in the apoHRP(0.22) gel/FePor(*y*) gel combination as the concentration of FePor increased from 0.47 to 1.0 mol% (Fig. S16b black dots and purple crosses).


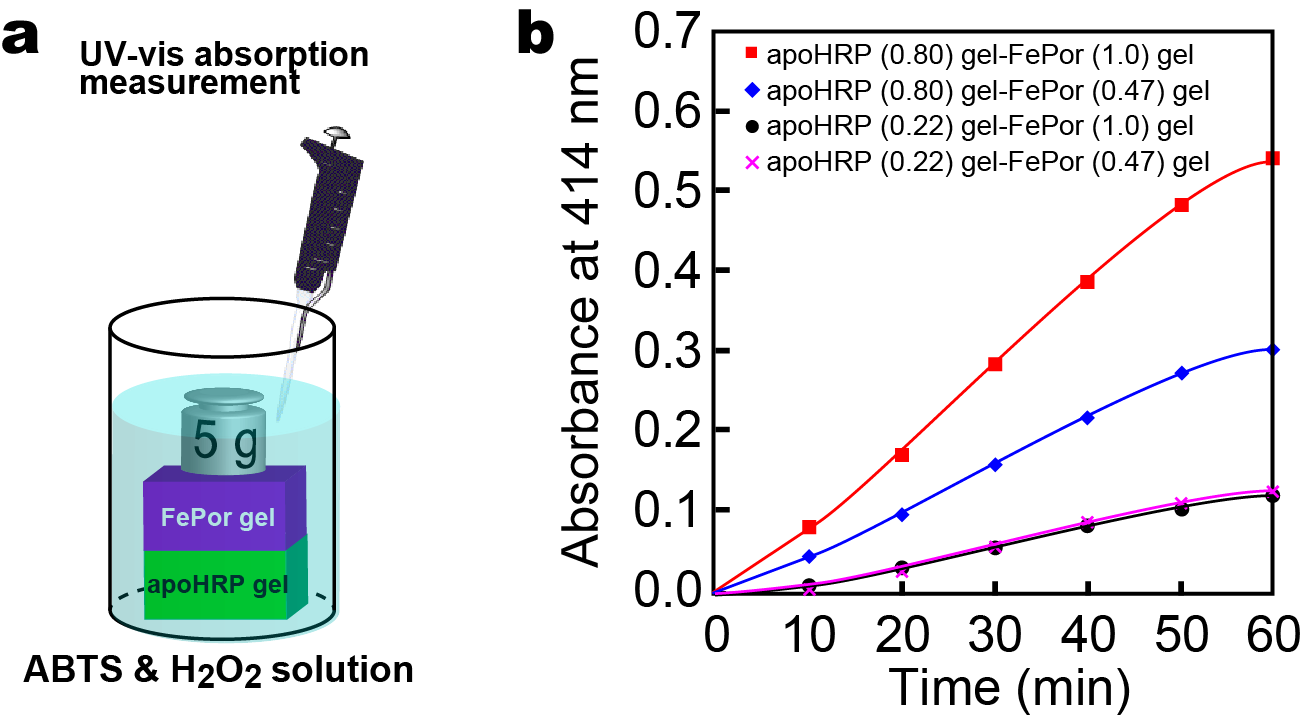


**Figure S16**. **a** Component drawing of the experiment to investigate oxidation of ABTS in the presence of the apoHRP gel/FePor gel combination with different mol% of apoHRP or FePor in the gel. **b** Oxidation of 0.5 mM ABTS in the presence of the following combinations: apoHRP(0.80) gel/FePor(1.0) gel (red squares), apoHRP(0.80) gel/FePor(0.47) gel (blue diamonds), apoHRP(0.22) gel/FePor(1.0) gel (black circles), and apoHRP(0.22) gel/FePor(0.47) gel (purple crosses).

**Switching the catalytic activity on and off via adhesion and separation of the apoHRP (0.8) and FePor (1.0) gels**

The apoHRP(0.8) and FePor(1.0) gels were soaked in a solution of 2.0 mM pyrogallol, 5.0 mM H_2_O_2_, 4% DMSO, and 50 mM sodium phosphate buffer (pH 7.0). The absorbance of the product was measured for 2 min.


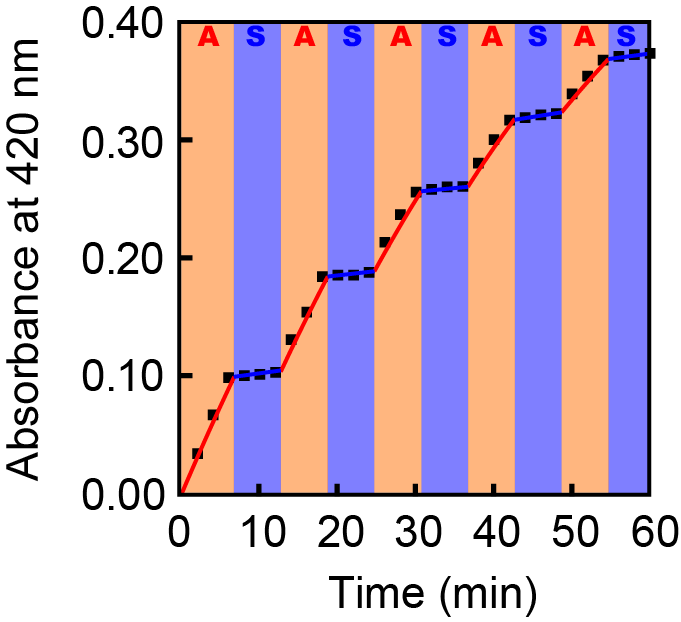


**Figure S17.** Oxidation of 2.0 mM pyrogallol in the presence of the apoHRP (0.80) gel/FePor (1.0) gel assembly (area A) and of the separated gels (area S).

**Movies**

**1. Supplementary Movie 1: Adhesion between the apoHRP and FePor gels**

The ApoHRP (1.0), FePor (1.0), and blank gels swelled in a solution containing 4% DMSO and 50 mM sodium phosphate buffer (pH 7.0). The apoHRP (1.0) gel was placed on top of the FePor (1.0) gel, and the stack was left undisturbed for 2 h at r.t. under humid conditions. Pieces of the same type of gel (e.g., the apoHRP (1.0) gel pieces) or pieces of the blank gel with the apoHRP (1.0) or FePor (1.0) gels were stacked under the abovementioned conditions. The combination of apoHRP (1.0) and FePor (1.0) gels formed a gel assembly, whereas the combination of apoHRP (1.0) and apoHRP (1.0) gels, FePor (1.0) and FePor (1.0) gels, apoHRP (1.0) and blank gels, and blank and FePor (1.0) gels did not form an assembly. The apoHRP gel/FePor gel assembly was sufficiently strong that it could be lifted with tweezers.

**2. Supplementary Movie 2:** The **apoHRP gel/FePor gel assembly is lifted using tweezers**

The apoHRP (1.0) and FePor (1.0) gels swelled in a solution containing 4% DMSO and 50 mM sodium phosphate buffer (pH 7.0). The FePor (1.0) and apoHRP (1.0) gels were stacked in an alternating manner, and the stack was left undisturbed for 2 h at r.t. under humid conditions. The apoHRP gel/FePor gel assembly could be lifted with tweezers.

**3. Supplementary Movie 3: Dissociation of the apoHRP gel/FePor gel assembly using apoHRP as a competitive molecule**

The apoHRP (1.0) gel/FePor (1.0) gel assembly was agitated for 20 minutes in a solution containing 4% DMSO and 50 mM sodium phosphate buffer (pH 7.0), and the gel assembly did not dissociate. The solution was replaced with a 350μM apoHRP aqueous solution with buffer. Then, the gel assembly dissociated using agitation for 20 minutes in apoHRP-containing buffer.

**4. Supplementary Movie 4: Catalytic oxidative reaction of ABTS expressed via adhesion of the apoHRP and FePor gels**

The apoHRP (1.0) gel was absorbed in a solution containing 0.5 mM ABTS, 5.0 mM H_2_O_2_, 4% DMSO, and 50 mM sodium phosphate buffer (pH 7.0). The FePor (1.0) gel swelled in a solution containing 4% DMSO and 50 mM sodium phosphate buffer (pH 7.0). ABTS is colourless. However, the oxidation product of ABTS via HRP and H_2_O_2_ is green. The FePor (1.0) gel was placed on top of ABTS and the H_2_O_2_-containing ApoHRP (1.0) gel. Then, a green oxidation product of ABTS appeared on the adhesive surface of the apoHRP (1.0) and FePor (1.0) gels.

**5. Supplementary Movie 5: The catalytic oxidative reaction of pyrogallol expressed via adhesion of the apoHRP to FePor gels**

The apoHRP (1.0) gel was absorbed in a solution containing 2.0 mM pyrogallol, 5.0 mM H_2_O_2_, 4% DMSO, and 50 mM sodium phosphate buffer (pH 7.0). The FePor (1.0) gel swelled in a solution containing 4% DMSO and 50 mM sodium phosphate buffer (pH 7.0). Pyrogallol is colourless. However, the oxidation product of pyrogallol via HRP and H_2_O_2_ is brown. The FePor (1.0) gel was placed on top of pyrogallol and the H_2_O_2_-containing ApoHRP (1.0) gel. Then, a brown product from the oxidation product of pyrogallol appeared on the adhesive surface of the apoHRP (1.0) and FePor (1.0) gels.
